# Efficient estimation of Pareto model: Some modified percentile estimators

**DOI:** 10.1371/journal.pone.0196456

**Published:** 2018-05-01

**Authors:** Sajjad Haider Bhatti, Shahzad Hussain, Tanvir Ahmad, Muhammad Aslam, Muhammad Aftab, Muhammad Ali Raza

**Affiliations:** 1 Department of Statistics, Government College University, Faisalabad, Pakistan; 2 Department of Statistics, Bahauddin Zakariya University, Multan, Pakistan; Southwest University, CHINA

## Abstract

The article proposes three modified percentile estimators for parameter estimation of the Pareto distribution. These modifications are based on median, geometric mean and expectation of empirical cumulative distribution function of first-order statistic. The proposed modified estimators are compared with traditional percentile estimators through a Monte Carlo simulation for different parameter combinations with varying sample sizes. Performance of different estimators is assessed in terms of total mean square error and total relative deviation. It is determined that modified percentile estimator based on expectation of empirical cumulative distribution function of first-order statistic provides efficient and precise parameter estimates compared to other estimators considered. The simulation results were further confirmed using two real life examples where maximum likelihood and moment estimators were also considered.

## 1. Introduction

Pareto distribution is widely applicable distribution in economics. It was initially introduced by Pareto [1] to represent the income distribution among individuals. It is most appropriate model for situations represented by 80–20 rule, that is, when 80% effect comes from 20% causes. Certainly, a large portion of wealth of society is used or owned by a small percentage of people. The Pareto model has wide application in economic studies as it plays a vital role in the investigation of several phenomena [2]. Although it is most widely used as an income model to define the allocation of wealth among individual units [3] but it is not limited to application only in economics as it has great utility in modeling number of casualties in earthquakes, forestry fire areas and oil & gas in different field sizes [4]. The applicability of Pareto model in real life phenomenon is evident in many studies like, [2,5–9]. Its generalized, exponentiated, modified, Kumaraswamy and transmuted versions have also been presented with real life applications [10–14].

The density function of Pareto distribution is given as

$$f(x\;;\alpha ,\beta )=\frac{\alpha \beta^{\alpha }}{x^{\alpha +1}},\;\beta \leq x\leq \infty ,\;\beta \;>\;0\;,\;\alpha \;>\;0,$$

where *β* is scale and *α* is shape parameter and it is denoted by *x* ~ Pareto(*β*, *α*).

**Fig 1:**
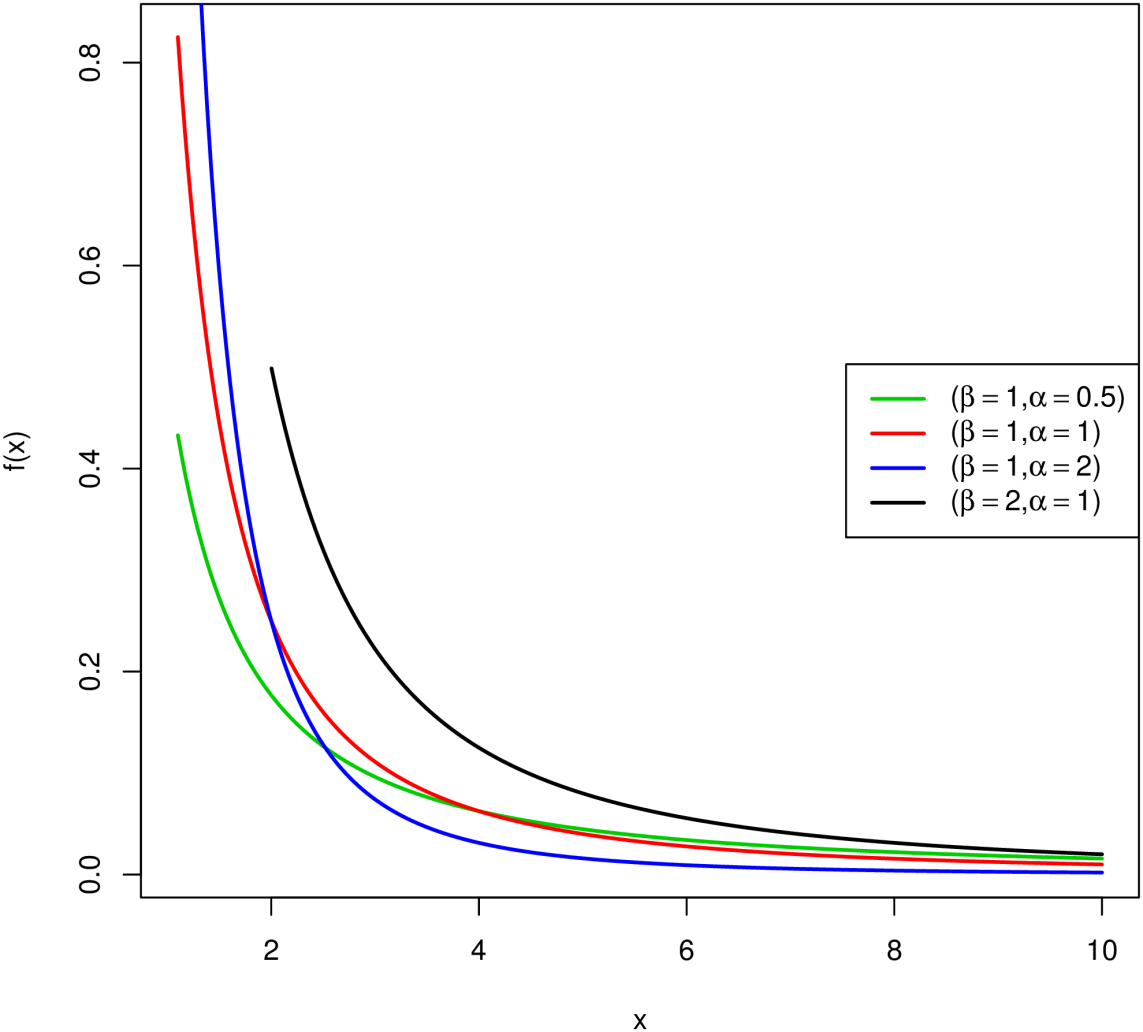
PDF of the Pareto distribution.

**Fig 2:**
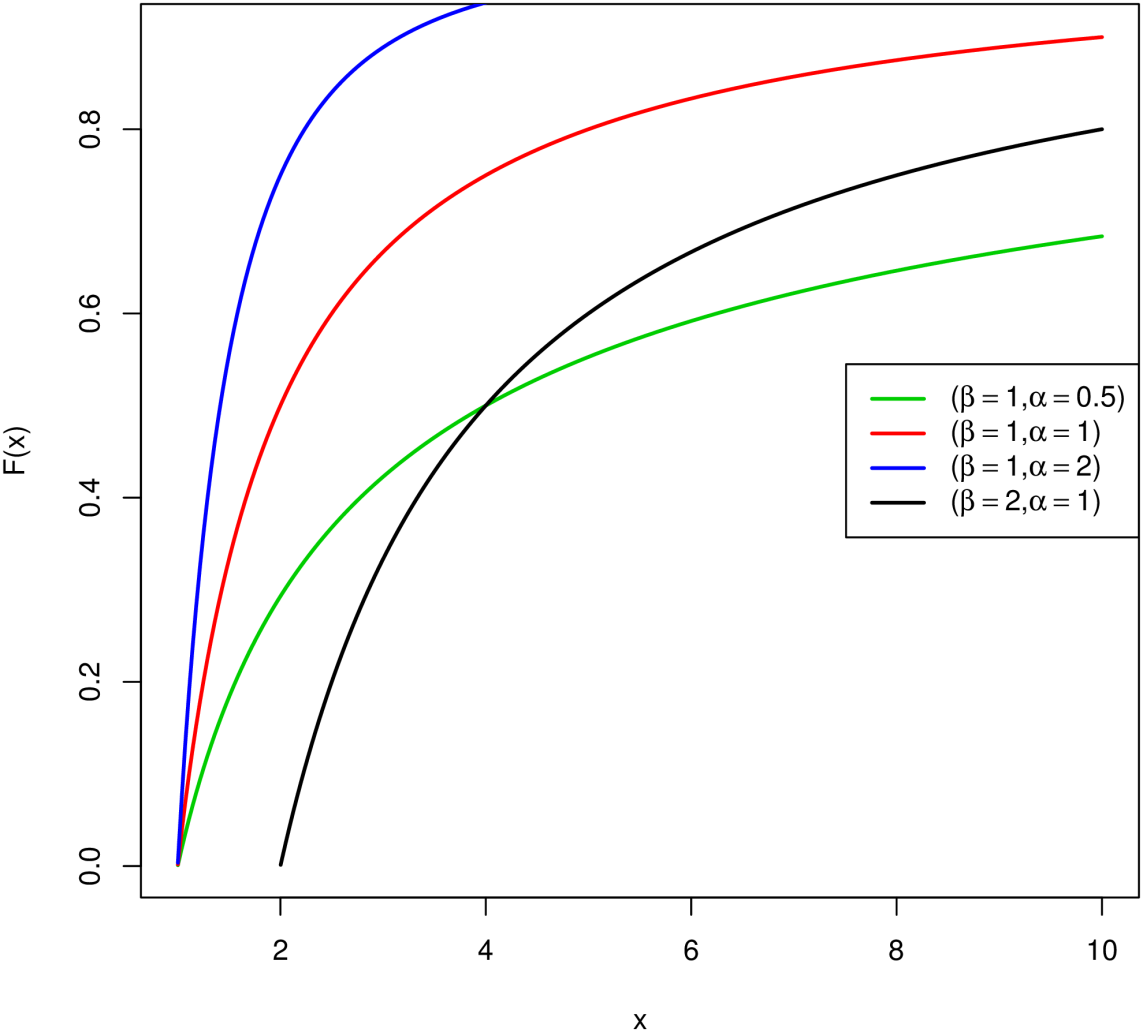
CDF of the Pareto distribution.

Shapes of Probability Density Function (PDF) and Cumulative Distribution Function (CDF) for different combinations of scale and shape parameters are shown in Figs 1 and 2, respectively.

In the literature concerning parameter estimation, different estimation strategies have been used for the Pareto distribution like Quandt [15] derived expressions for moments, maximum likelihood, percentile and least squares estimators. Kuldroff and Vannman [16] have proposed parameter estimation of the Pareto distribution by linear functions of order statistics. Afify [17] has employed distinct estimation procedures for parameter estimation of Pareto distribution and revealed that least squares estimators perform better in terms of root mean square error. Parameter estimation of the Pareto distribution have also been carried out using jackknife and minimum risk estimators [18]. Based on Monte Carlo simulation Lu and Tao [19] showed that maximum likelihood and weighted least squares methods were equally efficient.

Method of percentile estimation is in use for a long time. Parameters of different probability distributions have been estimated using percentile estimation method and found better or equally efficient to maximum likelihood and least squares techniques [20–22].

In the literature devoted to parameter estimation, different modifications have been proposed in standard estimation procedures. Modified maximum likelihood estimators and modified moment estimator have been introduced and found efficient than traditional estimators for different probability distributions like three-parameter log-normal [23–24], three-parameter Weibull [25], three-parameter Gamma [26], Rayleigh [27], two-parameter Exponential [28], and two-parameter Power Function [29].

Keeping in view the applicability and importance of the Pareto distribution in empirical studies, method of percentile estimation and superiority of modified estimators for different distributions in recent literature, present study is focused on deriving modified percentile estimators for Pareto distribution. The derived modifications have been compared with traditional percentile estimators through Monte Carlo simulation and two real life datasets.

## 2. Methodology

In the present work, we have suggested some modifications in percentile estimation method using median, geometric mean and expectation of first order statistic of empirical cumulative distribution function of the Pareto distribution. The modified estimators were compared with traditional percentile estimators.

### 2.1 Method of percentile estimation

Percentiles play an important role in descriptive statistics and their use is recommended for parameter estimation as well [30]. The principle is based on equating two values of cumulative distribution function with corresponding percentiles and then simultaneously solving resulting equations for unknown parameters. Following Marks [22], Zaka and Akhtar [29] and Sampath and Anjana [31], we have chosen P_25_ and P_75_ to be relatively more accurate in comparison to other pairs of percentiles.

### 2.2 Percentile estimator

Let *x*_1_, *x*_2_,…,*x*_n_ be a random sample of size *n* from Pareto distribution. The cumulative-distribution function of a Pareto distribution with shape and scale parameters *α* and *β*, respectively is,

$$F\left(x_{i}\right)=1-{\left(\frac{\beta }{x_{i}}\right)}^{\alpha }$$

Thus, using percentiles P_75_ and P_25_,

$$0.75=1-{\left(\frac{\beta }{P_{75}}\right)}^{\alpha },$$

$$\beta =P_{75}{\left(0.25\right)}^{\frac{1}{\alpha }}.$$ (1)

Similarly,

$$0.25=1-{\left(\frac{\beta }{P_{25}}\right)}^{\alpha },$$

$$\beta =P_{25}{\left(0.75\right)}^{\frac{1}{\alpha }}.$$ (2)

Solving Eqs (1) and (2) simultaneously for unknown parameters, we get the percentile estimators for *α* and *β* as,

$$\hat{\alpha }=\frac{\text{log}\left(3\right)}{\text{log}\left(P_{75}\right)-\text{log}\left(P_{25}\right)},$$ (3)

$$\hat{\beta }=P_{25}{\left(0.75\right)}^{{}^{\left(\frac{\text{log}\;\left(P_{75}\right)-\text{log}\left(P_{25}\right)}{\text{log}\left(3\right)}\right)}}.$$ (4)

Eqs (3) and (4) are the required percentile estimators of the Pareto distribution. For further reference, we name these estimators as PE.

### 2.3 Modified percentile estimator (I)

Our first modification in method of percentile estimation is based on replacing Eq (2) by median of the Pareto distribution as,

$$\tilde{X}=\beta 2^{\frac{1}{\alpha }}.$$ (5)

Rewriting Eq (1) as

$$\beta =P_{75}{\left(0.25\right)}^{\frac{1}{\alpha }}\cdot$$ (6)

Solving Eqs (5) and (6) simultaneously we get first modified percentile estimators for *α* and *β*,

$$\frac{\tilde{X}}{2^{{}^{\frac{1}{\alpha }}}}=P_{75}{\left(0.25\right)}^{\frac{1}{\alpha }}\;\;\;\;,$$

$$\frac{\tilde{X}}{P_{75}}=2^{{}^{\frac{1}{\alpha }}}{\left(0.25\right)}^{\frac{1}{\alpha }}\;\;\;,$$

$$\left[\text{log}\left(\tilde{X}\right)-\text{log}\left(P_{75}\right)\right]=\frac{1}{\alpha }\left[\text{log}\left(2\right)+\text{log}\left(0.25\right)\right]\;\;\;\;\;,$$

so,

$$\hat{\alpha }=\frac{\left[\text{log}\left(0.25\right)+\text{log}\left(2\right)\right]}{\left[\text{log}\left(\tilde{X}\right)-\text{log}\left(P_{75}\right)\right]}.$$ (7)

Putting value of $\hat{\alpha }$ from Eq (7) in Eq (6) we get estimate of *β* as

$$\hat{\beta }=\frac{\tilde{X}}{2^{\frac{1}{\left(\frac{\left[\text{log}\left(0.25\right)+\text{log}\left(2\right)\right]}{\left[\text{log}\left(\tilde{X}\right)-\text{log}\left(P_{75}\right)\right]}\right)}}},$$

$$\hat{\beta }=\frac{\tilde{X}}{2^{\left(\frac{\left[\text{log}\left(\tilde{X}\right)-\text{log}\left(P_{75}\right)\right]}{\left[\text{log}\left(0.25\right)+\text{log}\left(2\right)\right]}\right)}}\cdot$$ (8)

Eqs (7) and (8) provide expressions for first modified percentile estimators (PE-I, for further reference).

### 2.4 Modified percentile estimator (II)

Our second modified percentile estimator is based on replacing Eq (2) by Geometric Mean (GM) of the Pareto distribution.

$$\text{i}.\text{e}.\;\;GM=\beta e^{\frac{1}{\alpha }}$$ (9)

Rewriting Eq (1) as

$$\beta =P_{75}{\left(0.25\right)}^{\frac{1}{\alpha }}$$ (10)

Solving Eqs (9) and (10) simultaneously we get second modified percentile estimators for *α* and *β* as,

$$\frac{GM}{e^{{}^{\frac{1}{\alpha }}}}=P_{75}{\left(0.25\right)}^{\frac{1}{\alpha }}\;\;\;\;,$$

$$\frac{GM}{P_{75}}=e^{{}^{\frac{1}{\alpha }}}{\left(0.25\right)}^{\frac{1}{\alpha }}\;\;\;\;,$$

$$\left[\text{log}\left(GM\right)-\text{log}\left(P_{75}\right)\right]=\frac{1}{\alpha }\left[\text{log}\left(e\right)+\text{log}\left(0.25\right)\right]\;\;\;\;,$$

$$\hat{\alpha }=\frac{\left[\text{log}\left(0.25\right)+1\right]}{\left[\text{log}\left(G.M\right)-\text{log}\left(P_{75}\right)\right]}\;\;\cdot$$ (11)

Putting value of $\hat{\alpha }$ from Eq (11) in Eq (9) we get estimate of *β* as

$$\hat{\beta }=\frac{GM}{e^{\frac{1}{\frac{1+\text{log}\left(0.25\right)}{\text{log}\left(GM\right)-\text{log}\left(P_{75}\right)}}}}\;\;\;,$$

$$\hat{\beta }=\left(G.M\right)\;*\;\;{\text{e}}^{-\;\left(\frac{\left[\text{log}\left(G.M\right)-\text{log}\left(P_{75}\right)\right]}{\left[\text{log}\left(0.25\right)+1\right]}\right)}\cdot$$ (12)

Eqs (11) and (12) are the expressions for the second modified percentile (PE-II for further reference) estimators of the Pareto distribution.

### 2.5 Modified percentile estimator (III)

The third modified percentile estimator proposed is obtained by replacing Eq (2) by expectation of empirical cumulative distribution function of first order statistic of Pareto distribution.

Following [25,26,28,29] expectation of empirical CDF of first order statistic is defined as,

$$E\left[F\left(x_{\left(1\right)}\right)\right]=F\left(x_{\left(1\right)}\right)=\frac{1}{n+1}\cdot$$ (13)

So in case of the Pareto distribution,

$$\frac{1}{n+1}=1-{\left(\frac{\beta }{x_{\left(1\right)}}\right)}^{\alpha },$$

$${\left(\frac{\beta }{x_{\left(1\right)}}\right)}^{\alpha }=1-\frac{1}{n+1}\;\;\;,$$

$$\hat{\beta }=x_{\left(1\right)}{\left(\frac{n}{n+1}\right)}^{\left(\frac{1}{\alpha }\right)}\;\;\cdot$$ (14)

We have Eq (1) as

$$\beta =P_{75}{\left(0.25\right)}^{\frac{1}{\alpha }}.$$ (15)

Comparing Eqs (14) and (15),

$$P_{75}{\left(0.25\right)}^{\frac{1}{\alpha }}=x_{\left(1\right)}.{\left(\frac{n}{n+1}\right)}^{\frac{1}{\alpha }},$$

$$\text{log}\left(P_{75}\right)+\frac{1}{\alpha }\text{log}\left(0.25\right)=\text{log}\left(x_{\left(1\right)}\right)+\frac{1}{\alpha }\text{log}\left(\frac{n}{n+1}\right)\;\;\;,$$

$$\frac{1}{\alpha }\text{log}\left(0.25\right)-\frac{1}{\alpha }\text{log}\left(n\right)+\frac{1}{\alpha }\text{log}\left(n+1\right)=\text{log}\left(x_{\left(1\right)}\right)-\text{log}\left(P_{75}\right),\;\hat{\alpha }=\frac{\left[\text{log}\left(0.25\right)-\text{log}\left(n\right)+\text{log}\left(n+1\right)\right]}{\left[\text{log}\left(x_{\left(1\right)}\right)-\text{log}\left(P_{75}\right)\right]}\;\;\cdot$$ (16)

Eqs (14) and (16) give algebraic expressions for third modified percentile estimators (PE-III for further reference) of parameters of the Pareto distribution.

### 2.6 Performance indices

In order to compare efficiency and accuracy of different estimators, Total Mean Square Error (TMSE) and Total Relative Deviation (TRD) were used as performance indices. These measures are frequently used as performance criterion when different estimators (or estimation strategies) are compared through Monte Carlo simulation [28,29,32–39].

These performance indices are defined as,

$$TMSE=\frac{\sum_{r=1}^{\text{R}EP}\left[{\left({\hat{\beta }}_{r}-\beta \right)}^{2}+{\left({\hat{\alpha }}_{r}-\alpha \right)}^{2}\right]}{REP}\;\;\;\;=\;\;\;\;MSE\left(\hat{\beta }\right)\;\;+\;\;MSE\left(\hat{\alpha }\right)\cdot$$ (17)

and

$$TRD=\left|\frac{E(\hat{\alpha })-\alpha }{\alpha }\right|+\left|\frac{E(\hat{\beta })-\beta }{\beta }\right|,$$ (18)

where *α* and *β* are the true parameters, REP is the number of replications while $\hat{\alpha }$ and $\hat{\beta }$ are the parameter estimates.

As true parameters are unknown in real life data set, total mean square error and total relative deviation cannot be used for assessing performance of estimators in such cases. Therefore, we have used Mean Absolute Error (MAE), Mean Absolute Percentage Error (MAPE), Root Mean Square Error (RMSE) and Root Mean Square Percentage Error (RMSPE) as performance measures for comparison among different estimators. These measures are defined as,

$$MAE\;=\;\frac{\sum_{i=1}^{n}\left|S\left(x_{i}\right)-\hat{F}\left(x_{i}\right)\right|}{n}\cdot$$ (19)

$$MAPE\;=\;\frac{\sum_{i=1}^{n}\left|\frac{S\left(x_{i}\right)-\hat{F}\left(x_{i}\right)}{S\left(x_{i}\right)}\right|}{n}*100\;\;\;\cdot$$ (20)

$$RMSE\;=\;\sqrt{\frac{\sum_{i=1}^{n}{\left\{S\left(x_{i}\right)-\hat{F}\left(x_{i}\right)\right\}}^{2}}{n}}\;\;\cdot$$ (21)

$$RMSE\;=\;\sqrt{\frac{\sum_{i=1}^{n}{\left\{\frac{S\left(x_{i}\right)-\hat{F}\left(x_{i}\right)}{S\left(x_{i}\right)}\right\}}^{2}}{n}}*100\;\;\;\cdot$$ (22)

where *S*(*x*_*i*_) is sample (observed) distribution function and $\hat{F}\left(x_{i}\right)$ is expected distribution function which are respectively defined as,

$$S\left(x_{i}\right)\;=\frac{\text{number}\;\text{elements}\;\text{the}\;\text{sample}\leq x}{n}=\frac{1}{n}\sum_{i=1}^{n}1_{x_{i}\leq x}$$

and $\hat{F}\left(x_{i}\right)=\;1-{\left(\frac{\hat{\beta }}{x_{i}}\right)}^{\hat{\alpha }}$ with parameter estimates ($\hat{\alpha }$ and $\hat{\beta }$) form any particular method.

## 3. Monte Carlo simulation

A Monte Carlo simulation study was performed to compare the proposed modified percentile estimators with traditional percentile estimation. This comparison was carried out by taking random samples of different sizes (n = 20, 50, 100, 200, 500 and 1000) with different pairs of parameter values (*β*, *α*) = (1, 0.5), (1, 1), (1, 2), (2, 1).

For any combination of true parameters (*β* and *α*), Monte Carlo simulation was performed by carrying out following steps in R-language [40].

- A sample of n uniform random numbers was generated in interval [0,1].
  i.eU~Unif[0,1]
- Uniform random numbers were converted in Pareto random variables by following relation.
  $$x=\frac{\beta }{{\left(1-U\right)}^{\frac{1}{\alpha }}}$$
- The process in above steps was repeated 10000 times.

## 4. Results and discussion

Tables 1–4 present the results of Monte Carlo simulation study carried out for numerical evaluation of the estimators considered for different sample sizes and different parameter combinations.

**Table 1:** Comparison of PE and modified PE for *β* = 1, *α* = 0.5.

*n*	Method	$E\left(\hat{\beta }\right)$	$E\left(\hat{\alpha }\right)$	TMSE	TD
20	PE	1.177512	0.581113	0.266123	0.339737
	PE-I	1.454896	0.646239	1.696595	0.747374
	PE-II	1.839641	1.858232	10974.32	3.556105
	PE-III	1.008379	0.539913	0.040392	0.088205
50	PE	1.068149	0.534724	0.068059	0.137597
	PE-I	1.17388	0.557861	0.37154	0.289601
	PE-II	1.353715	0.638099	19.03515	0.629914
	PE-III	1.001518	0.519449	0.011083	0.040415
100	PE	1.034997	0.517485	0.030121	0.069967
	PE-I	1.090907	0.528881	0.161391	0.148668
	PE-II	1.168791	0.555963	0.50695	0.280717
	PE-III	1.000106	0.509616	0.004604	0.019339
200	PE	1.018252	0.508912	0.014463	0.036076
	PE-I	1.045447	0.514297	0.072335	0.074042
	PE-II	1.089797	0.526584	0.202829	0.142964
	PE-III	1.000175	0.504883	0.002135	0.009941
500	PE	1.008184	0.503561	0.005383	0.015306
	PE-I	1.017841	0.505505	0.027563	0.028852
	PE-II	1.035348	0.509962	0.070451	0.055272
	PE-III	1.000039	0.501819	0.000816	0.003676
1000	PE	1.004332	0.502336	0.002682	0.009004
	PE-I	1.010203	0.503484	0.013382	0.017172
	PE-II	1.019159	0.505688	0.033011	0.030536
	PE-III	1.000024	0.501422	0.000401	0.002867

**Table 2:** Comparison of PE and modified PE for *β* = 1, *α* = 1.

*n*	Method	$E\left(\hat{\beta }\right)$	$E\left(\hat{\alpha }\right)$	TMSE	TRD
20	PE	1.073072	1.177465	0.248254	0.250536
	PE-I	1.13686	1.305223	0.731095	0.442083
	PE-II	1.190234	13.56927	2016418	12.75951
	PE-III	1.00298	1.094674	0.117658	0.097654
50	PE	1.027124	1.066142	0.069593	0.093265
	PE-I	1.051458	1.109918	0.192625	0.161376
	PE-II	1.083264	1.233346	70.59511	0.31661
	PE-III	1.000803	1.036014	0.036107	0.036817
100	PE	1.014099	1.033143	0.03049	0.047242
	PE-I	1.029883	1.056482	0.083419	0.086365
	PE-II	1.048096	1.304341	362.9731	0.352436
	PE-III	0.999921	1.018084	0.016678	0.018163
200	PE	1.006765	1.017074	0.014677	0.023839
	PE-I	1.013689	1.027462	0.037971	0.04115
	PE-II	1.022471	1.050664	0.097214	0.073136
	PE-III	1.000147	1.00994	0.008391	0.010088
500	PE	1.002691	1.006199	0.005653	0.00889
	PE-I	1.005496	1.010362	0.01465	0.015859
	PE-II	1.010301	1.019627	0.034065	0.029928
	PE-III	0.999989	1.003309	0.003229	0.00332
1000	PE	1.001036	1.002801	0.002721	0.003837
	PE-I	1.002577	1.00494	0.007083	0.007517
	PE-II	1.004016	1.008624	0.015701	0.01264
	PE-III	0.999991	1.001597	0.001564	0.001606

**Table 3:** Comparison of PE and modified PE for *β* = 1, *α* = 2.

*n*	Method	$E\left(\hat{\beta }\right)$	$E\left(\hat{\alpha }\right)$	TMSE	TRD
20	PE	1.032774	2.356148	0.843406	0.210848
	PE-I	1.052433	2.622239	2.321268	0.363552
	PE-II	1.058361	2.805987	6327.576	0.461354
	PE-III	1.001137	2.189173	0.450276	0.095724
50	PE	1.012796	2.136993	0.239532	0.081293
	PE-I	1.022312	2.235901	0.54974	0.140262
	PE-II	1.027095	2.433643	108.4377	0.243917
	PE-III	1.000107	2.073907	0.146156	0.037061
100	PE	1.005777	2.064917	0.103872	0.038235
	PE-I	1.009939	2.109024	0.217326	0.064451
	PE-II	1.013769	2.243651	2.495466	0.135594
	PE-III	1.00005	2.035985	0.067343	0.018043
200	PE	1.003326	2.033866	0.049188	0.020259
	PE-I	1.005871	2.056703	0.098455	0.034223
	PE-II	1.007418	2.102194	0.245855	0.058515
	PE-III	1.000039	2.018416	0.032966	0.009247
500	PE	1.001151	2.01088	0.018004	0.006591
	PE-I	1.00144	2.017816	0.035774	0.010347
	PE-II	1.002177	2.034162	0.07737	0.019259
	PE-III	0.999995	2.005349	0.012406	0.002679
1000	PE	1.000385	2.005086	0.00912	0.002928
	PE-I	1.000651	2.008925	0.017945	0.005113
	PE-II	1.001093	2.017253	0.037491	0.00972
	PE-III	1	2.002843	0.006299	0.001422

**Table 4:** Comparison of PE and modified PE for *β* = 2, *α* = 1.

*n*	Method	$E\left(\hat{\beta }\right)$	$E\left(\hat{\alpha }\right)$	TMSE	TRD
20	PE	2.141995	1.177234	0.361573	0.248231
	PE-I	2.270806	1.308429	1.313734	0.443832
	PE-II	2.360906	1.636639	1427.492	0.817092
	PE-III	2.005043	1.093012	0.12478	0.095533
50	PE	2.062342	1.07597	0.112838	0.107141
	PE-I	2.125702	1.128606	0.408916	0.191457
	PE-II	2.19821	1.269502	30.75832	0.368607
	PE-III	2.000992	1.04154	0.040189	0.042036
100	PE	2.030303	1.034942	0.048574	0.050094
	PE-I	2.06161	1.058565	0.182036	0.089371
	PE-II	2.099942	1.112929	1.408045	0.162899
	PE-III	2.000317	1.019028	0.017631	0.019187
200	PE	2.015067	1.016709	0.02239	0.024243
	PE-I	2.031228	1.028304	0.08437	0.043918
	PE-II	2.048506	1.050127	0.203557	0.07438
	PE-III	2.000178	1.008988	0.008096	0.009077
500	PE	2.006247	1.006946	0.008637	0.010069
	PE-I	2.012911	1.01165	0.033774	0.018106
	PE-II	2.020555	1.019767	0.076773	0.030045
	PE-III	2.000022	1.003743	0.003112	0.003754
1000	PE	2.003247	1.003992	0.004365	0.005616
	PE-I	2.006534	1.006228	0.016464	0.009495
	PE-II	2.011257	1.01054	0.037368	0.016169
	PE-III	1.999969	1.002341	0.001592	0.002356

Results from Table 1 (for *β* = 1; *α* = 0.5) show that modified percentile estimator PE-III (which is based on expectation of empirical cumulative distribution function of first-order statistic) more accurately estimated true parameters compared to traditional percentile estimator and other modified percentile estimators (based on median and geometric mean). From these results, under total mean square error criterion, third modified percentile estimator provided more efficient parameter estimates for all sample sizes as it has lower values of total mean square error values than other competing estimators. Based on second performance criterion, total relative deviation, it is interesting to note that for all samples sizes we come to same conclusion that third modified percentile estimators is more efficient among all estimation strategies considered. It is worth noticing that traditional percentile estimator is second best choice after third modified percentile estimator.

Concerning literature devoted to modified estimators, our results coincide with other studies favouring use of modified maximum likelihood, moment and percentile estimation for different probability distributions [25–29].

Avoiding repetition, it can be stated that PE-III provides more efficient and accurate estimates of parameters than other estimators considered for all sample size for parameter combinations (*β* = 1, *α* = 1), (*β* = 1, *α* = 2) and (*β* = 2, *α* = 1) presented in Tables 2–4, respectively.

Moreover, from results in Tables 1–4, it can also be observed that modified estimator PE-II (based on geometric mean) is worst performer in terms of both performance indicators. However, its performance gets better gradually with increasing sample size. The reason behind its poor performance in small samples may be that geometric mean is influenced by extreme values which is common in heavy tailed distributions like Pareto.

## 5. Real life examples

In addition to numerical evaluation of proposed estimators through simulation study, the modified percentile estimators were applied on two real life data sets.

For comparison purpose, we have also used maximum likelihood and moment estimators of Pareto distribution. The Maximum Likelihood (ML) estimator of *α* and *β* are,

$${\hat{\alpha }}_{ML}=\frac{n}{\sum_{i=1}^{n}\text{ln}x_{i}-n\text{ln}\beta },$$ (23)

$${\hat{\beta }}_{ML}=x_{\left(1\right)}.$$ (24)

Similarly, the estimators from Method of Moments (MM) are

$${\hat{\alpha }}_{MM}=1+\sqrt{1+\frac{{\bar{X}}^{2}}{S^{2}}},$$ (25)

$${\hat{\beta }}_{MM}=\frac{\sqrt{S^{2}+{\bar{X}}^{2}}}{S+\sqrt{S^{2}+{\bar{X}}^{2}}}\bar{X}.$$ (26)

**Example 1**: First example is taken from Clark [9], it consists of 21 observations about number of deaths in major earthquakes during 1900–2011 as published by the United States Geological Survey. The results from application of proposed estimators on example 1 are presented in Table 5.

**Table 5:** Comparison of estimators for example 1.

Method	$\hat{\beta }$	$\hat{\alpha }$	MAE	MAPE	RMSE	RMSPE
ML	20085	0.903376	0.038434	14.42816	0.047099	27.24039
MM	53146.93	2.443539	2.35963	2061.182	3.822201	5120.551
PE	21348.21	0.845553	0.054456	27.54816	0.065346	55.94947
PE-I	22727.27	0.879118	0.072681	42.04517	0.089363	88.84464
PE-II	13071.23	0.650822	0.085454	49.33647	0.102901	104.4611
PE-III	18933.4	0.787868	0.031231	6.586841	0.04287	8.599407

Results from Table 5 clearly indicate the superiority of PE-III in comparison to other percentile based estimators as well as to maximum likelihood and moment estimators. All four performance measures have smaller values for PE-III than other estimators.

**Example 2**: Second data set is taken from Beirliant et al. [41] consisting of 142 values of fire damage claims (in 1000’s of Norwegian Krones) in Norway during 1975. This data set have also been used by some other studies focusing on Pareto distribution [3,42,43].

Table 6 shows that based on three performance indices, third modified percentile estimator (PE-III) is better than traditional percentile (PE), maximum likelihood (ML), moment (MM) and other modified percentile estimators (PE-I, PE-II). However, maximum likelihood estimation performs slightly better that PE-III in terms of mean absolute error.

**Table 6:** Comparison of estimators for example 2.

Method	$\hat{\beta }$	$\hat{\alpha }$	MAE	MAPE	RMSE	RMSPE
ML	500	1.19403	0.014455	6.980319	0.019478	17.45636
MM	2685.672	2.007348	10.58988	9363.318	13.59856	24469.07
PE	489.6091	1.250545	0.017239	8.067387	0.021113	14.84931
PE-I	559.4344	1.421501	0.057218	57.14853	0.080562	162.745
PE-II	604.7408	1.54486	0.10874	109.9473	0.153703	307.4798
PE-III	497.241	1.268241	0.015093	6.736636	0.018156	13.48124

## 6. Conclusion

Three modified percentile estimators are proposed for parameter estimation of the Pareto distribution. The modifications are based on median, geometric mean and expectation of empirical cumulative distribution function of first order statistic of Pareto distribution. Newly proposed estimators are compared with the traditional percentile estimators via Monte Carlo simulation and performance of modified percentile estimator based on expectation of empirical cumulative distribution function of first-order statistic is found better than traditional and other modified percentile estimators in terms of mean square error and total relative deviation. The Monte Carlo simulation results were further corroborated by application of proposed estimators on two real-life examples. From real life applications, it is shown that modified percentile estimator based on expectation of empirical cumulative distribution function of first order statistic performs better than not only other percentile based estimators but also maximum likelihood and moment estimators. Considering results from simulation and real data applications, use of modified percentile estimation can be recommended for estimating parameters of the Pareto distribution.

## Supporting information

S1 Data MINIMAL DATA.xlsx. (XLSX)
